# Physical traits of supercompetitors in cell competition

**DOI:** 10.1098/rsif.2025.0638

**Published:** 2025-10-08

**Authors:** Logan C. Carpenter, Shiladitya Banerjee

**Affiliations:** ^1^ Carnegie Mellon University, Pittsburgh, PA, USA; ^2^ Georgia Institute of Technology, Atlanta, GA, USA

**Keywords:** cell competition, cell growth, tissue homeostasis, apoptosis, cellular Potts model, cancer

## Abstract

Cell competition is a fitness control mechanism in tissues, where less fit cells are eliminated to maintain tissue homeostasis. Two primary mechanisms of cell competition have been identified: contact-dependent apoptosis and mechanical stress-induced competition. While both operate in tissues, their combined impact on population dynamics is unclear. Here, we present a cell-based computational model that integrates cellular mechanics with proliferation, contact-induced apoptosis and mechanically triggered apoptosis to investigate competition between two distinct cell types. Using this framework, we systematically examine how differences in physical traits—such as stiffness, adhesion and crowding sensitivity—govern competitive outcomes. Our results show that apoptosis rates alone are insufficient to predict cell fate; differences in proliferation and contact inhibition play equally important, context-dependent roles. Notably, we find that increased cell stiffness can confer a fitness advantage, enabling stiffer cells to outcompete softer neighbours. However, cells with reduced stiffness can become ‘soft’ supercompetitors if they exhibit faster growth and lower sensitivity to crowding. We also demonstrate that colony size critically influences competition: a minimum size is required for mutant expansion, below which elimination becomes stochastic. This stochastic clearance is driven by a protrusive instability in the interface between two cells that promotes invasion of the supercompetitors.

## 1. Introduction

Cell competition is a quality control process in tissues, where less fit cells are eliminated by their fitter neighbours [1–3]. This phenomenon plays a key role in tissue development, maintenance of tissue homeostasis and in the progression of cancer [4]. First discovered in *Drosophila* [5], cell competition was initially thought to act as a homeostatic mechanism, removing mutant cells with lower proliferative fitness. Later studies revealed mutants with a competitive advantage over wild-type cells that drive the elimination of wild-type cells [6]. A critical question is: what are the biophysical traits of mutations that provide a competitive advantage or disadvantage over normal cells?

Two primary modes of cell competition have been identified in previous studies: mechanical competition that can act over a distance [1,7–9], and contact-based competition, which directly affects the fate of neighbouring cells through intercellular signalling [10–14]. Mechanical competition is typically driven by differences in growth rates between ‘winner’ and ‘loser’ cell types, creating mechanical stress on the losers, ultimately leading to their apoptosis or extrusion [7,15]. However, additional cellular factors such as cytoskeletal rigidity, membrane tension, preferred cell density and cell–cell adhesion can also influence a cell’s ability to resist mechanical forces. A related determining factor in mechanical competition is contact inhibition of proliferation [16], which describes how cell growth and proliferation are inhibited in crowded environments [17–21]. Inducing mutations that downregulate the cellular contact inhibition has been shown to produce oncogenic-like supercompetitors [22–24]. While contact-based signalling depends critically on heterotypic cell–cell interactions [25–27], mechanical competition can emerge independently of such contacts, driven by differential growth or physical properties alone [1,7]. Both mechanical and contact-based elimination processes can coexist in tissues [28], but their relative contributions to population size and cell survival are not well understood. This presents an experimental challenge, given current limitations in isolating the diverse biophysical factors, and in connecting single-cell behaviours to population-level dynamics [29]. To address this, theoretical models can provide valuable insights into how cell-level properties and processes of proliferation and death influence tissue-scale outcomes in cell competition.

Previous theoretical studies on cell competition have considered predator–prey interactions in continuous models [30,31], the impact of mechanical stress on cell growth and apoptosis in continuum models [7,15,32,33], and cell-based models using the framework of vertex and cellular Potts models [34–37]. Cell-based models are particularly useful for linking single-cell behaviours to population-level dynamics [29]. Existing models have examined how factors like cell mechanics [35,38], cell–cell adhesion [35,39], contractility [40] and death signalling [36] influence the outcomes of cell competition between ‘winner’ and ‘loser’ phenotypes. However, these models have not addressed the combined effects of mechanical and contact-based apoptosis on population survival.

While prior work, including our own [35], has modelled the behaviour of specific mutants such as *scribble* by jointly altering multiple cellular traits (e.g. increased apoptosis sensitivity and reduced proliferation), the present study takes a complementary, reductionist approach. We aim to systematically isolate the role of individual physical or regulatory traits, such as compressibility or crowding sensitivity, in driving competitive fitness. This approach allows us to identify minimal conditions that are sufficient to confer a competitive advantage, providing a framework for disentangling the complex interplay of traits often co-modulated in genetic mutants.

We advance our recently developed cell-based model for epithelial tissue growth and homeostasis [41] to now simulate multiple cell types and their elimination via contact-based and mechanical apoptosis. Our model comprises two integrated layers: one that captures the physical dynamics of tissues and another that determines cellular fate through probabilistic decision-making rules. The physical layer employs the framework of the cellular Potts model [42], while the decision-making layer establishes rules for cell-cycle regulation, contact inhibition of proliferation and cell elimination via apoptosis and extrusion. Unlike previous cellular competition models, we do not assign winner or loser fates to cell types, but rather determine the cellular traits that lead to the emergence of losers or winners in cell competition. Our approach implements contact inhibition through crowding-induced suppression of cellular growth rates and considers the interplay between mechanical and contact-based cell competition. Additionally, we incorporate biphasic cell-cycle progression that allows for tunable growth and introduce distinct probabilities for apoptosis based on cell density and the proportion of heterotypic circumferential contact. Collectively, our model integrates feedback between mechanical pressure and the rates of cell growth, division and apoptosis within the context of cell competition.

Using this model, we predicted key characteristics of supercompetitive cell colonies. These include both stiffer cells that resist compression and outcompete their neighbours, as well as softer cells that can win when endowed with faster cell-cycle progression and reduced sensitivity to crowding. We also identify a critical colony size necessary for invasion. The size-dependent elimination of fitter mutants underscores the crucial role of fitness monitoring within wild-type epithelial tissues. Essentially, the earlier the competitive mechanisms activate to eliminate a highly proliferative mutant, the better the chances for the wild-type tissue to prevail. Consequently, mutants with shorter cell cycles have a higher likelihood of reaching the critical size. Interestingly, we discovered that mutant cells that are softer than their wild-type counterparts, can succeed by reducing their sensitivity to crowding. Taken together, our works reveals the intricate interplay between mechanical and decision-making elements of cells in regulating population growth, survival and extinction.

## 2. Cell competition modelling framework

**Figure 1.**
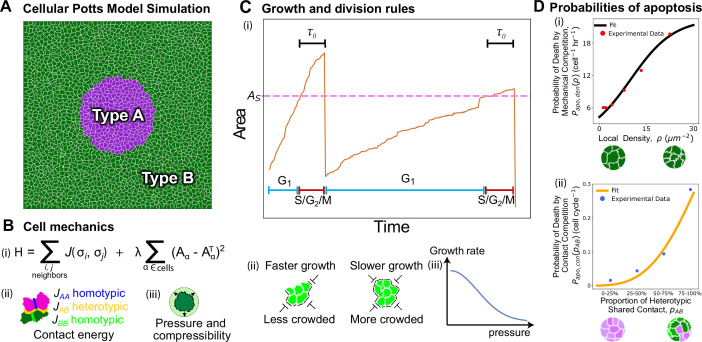
Overview of the computational model. (A) Snapshot at the beginning of a simulation. The purple cells, type A, represent the mutant population. The green cells, type B, represent the wild-type population; throughout this study, the default parameters defining these cell populations remain constant except where changes to type A are explicitly stated. (B) (i) The Hamiltonian used within the cellular Potts model to determine how the system mechanics evolves. (ii) Diagram of contact energies for heterotypic and homotypic contact. (iii) Schematic depicting forces promoting growth due to mechanical pressure and compressibility λ of cells. (C) Rules for cell growth and division. (i) A plot representing a cell's area trajectory over two cell cycles. The cell remains in the G1 phase until its area exceeds the sizer threshold at which point it transitions to the S/G2/M phase for a duration equal to the timer threshold, $\tau_{0}$. (ii) and (iii) The diagrams depict (ii) cells with space to expand into growing faster and (iii) cells with no space to grow into growing slower. The qualitative effects of these two scenarios for crowding is represented in the above area plot. Growth dynamics are determined by changing the target area of the cell, $A^{T}$, at a rate that decreases with increasing pressure on the cell. (D) Best fit curves for the probability of apoptosis as a function of (i) local density (equation (2.5); data from [35]) and (ii) the proportion of heterotypic shared contact (equation (2.6); data from [12]).

To study the dynamics of cell competition on the scale of individual cells, we implemented a two-layered computational model: (i) a physical layer that simulates the mechanics of the tissue via energy minimization, and (ii) a decision-making layer that simulates cell-autonomous dynamics such as growth, mitosis and apoptosis (figure 1). This framework allows us to characterize individual cell types by their expressed behaviours and physical characteristics informed by experiments [28].

### 2.1. Physical dynamics

The physical layer of the model is simulated using the cellular Potts model (CPM) [42], a computational framework widely used in the study of cell behaviours and tissue morphogenesis [43] (appendix A). The CPM simulates a cell on a discrete lattice as a collection of lattice-sites assigned a unique positive integer identifier $n_{id}$, with $n_{id}=0$ used for the extracellular medium (figure 1A). The mechanical energy of the system is given by


(2.1) $$\begin{array}{r} E=\sum_{\begin{array}{c} i,j\\ \text{neighbours: }n_{i}\neq n_{j} \end{array}}J_{ij}\left(\sigma_{i},\sigma_{j}\right)+\sum_{\alpha \in \text{cells}}\lambda_{\alpha }{\left(A_{\alpha }-A_{\alpha }^{T}\right)}^{2}\;, \end{array}$$

where the first sum captures the contact energy arising from cell–cell or cell–medium adhesion, and the second term describes the area elastic energy (figure 1B). The cell type at lattice site i is given by $\sigma_{i}$, which can take on values $\sigma_{i}=\left\{A:\text{type A},B:\text{type B},M:\text{Medium}\right\}$ (figure 1A). Then the contact energy between lattice sites i and j can take one of five possible values $J_{ij}(A,A)=J_{AA}$, $J_{ij}(A,B)=J_{AB}$, $J_{ij}(B,B)=J_{BB}$, $J_{ij}(A,M)=J_{AM}$ or $J_{ij}(B,M)=J_{BM}$. Due to energy minimization, a lower value of contact energy implies a greater adhesion strength. As a default, we assumed the greatest homotypic adhesion between type B cells (wild-type), and a lower homotypic adhesion between type A cells (mutants), and the least adhesion between cells and the medium $J_{BB}<J_{AB}<J_{AA}<J_{BM}=J_{AM}$. The second sum in equation (2.1) iterates over each cell in the simulation to calculate their area elastic energies. For a given cell α, this is done by penalizing deviations of the true cell area $A_{\alpha }$ from its preferred (target) value $A_{\alpha }^{T}$, multiplied by the compressional modulus $\lambda_{\alpha }=\{\lambda_{A}:\alpha \text{ is type A},\;\lambda_{B}:\alpha \text{ is type B}\}$. Both adhesion and elastic energy parameters are varied in simulations. We note that our model does not include a perimeter constraint term, which is sometimes used in alternative implementations of the CPM to represent cortical contractility [44]. In our case, interfacial contractility and adhesion are already captured via the contact energy term, and we avoid introducing additional parameters not directly constrained by experimental data.

The physical layer progresses in time by minimizing the mechanical energy E via implementation of the Metropolis–Hastings algorithm [45,46]. This is done by a semi-stochastic flipping of the cell identity at individual lattice sites; for instance, the algorithm considers adjacent sites i and j (with distinct cell identities $n_{i}\neq n_{j}$), and calculates the change in system energy resulting from the change of cell identity at i to $n_{j}$ ($\Delta E=E_{i\to n_{j}}-E_{i\to n_{i}}$). The probability of accepting the flip is given by


(2.2) $$\begin{array}{lcr} & \begin{array}{r} P(n_{i}\to n_{j})=\left\{ \begin{array}{cc} 1 & \text{if }\Delta E\leq 0\\ e^{-\Delta E/k_{B}T} & \text{otherwise} \end{array}\right., \end{array} & \end{array}$$

where $k_{B}$ is the Boltzmann constant and T is an effective temperature that reflects active fluctuations in cell behaviour (e.g. cytoskeletal activity or shape variability). Increasing T leads to greater cell boundary roughness, whereas low T enforces more compact and regular cell shapes. We chose a value of T such that cells exhibit moderate interface fluctuations without fragmenting or becoming overly jagged (see appendix A). A complete time step of the algorithm consists of x lattice copy attempts, where x is the number of lattice sites in the simulation ($x=1500^{2}$ in this work).

### 2.2. Decision-making rules

The physical layer of the CPM is coupled to a decision-making layer that implements various cell-autonomous rules between each simulation timestep, such as altering the cell’s target area, progressing the cell cycle phase, executing mitosis, initiating apoptosis and performing extrusion of cells. For a given cell α, growth dynamics are governed by increasing the cell’s target area over time as [35,41] (figure 1C),


(2.3) $$\frac{\text{d}A_{\alpha }^{T}}{\text{d}t}=G_{\alpha }e^{-k_{\alpha }(A_{\alpha }-A_{\alpha }^{T}{)}^{2}},\;\text{if}\;A_{\alpha }<A_{\alpha }^{T},$$

where $G_{\alpha }$ represents the uncrowded growth rate (or growth rate in isolation) of cell α, when $A_{\alpha }\approx A_{\alpha }^{T}$, and k is a phenomenological parameter that captures cellular sensitivity to crowding or contact inhibition of proliferation. The α subscript on G and k is to distinguish the dependence of these values on cell type. When a cell lacks the physical space to grow, then the cellular pressure $\propto -\Delta A_{\alpha }=-\left(A_{\alpha }-A_{\alpha }^{T}\right)$ will increase, which in turn causes an exponential decay of the rate of growth in proportion to k. Furthermore, crowding leads to a decreased rate of division as the cell cycle progression from G1 to S/G2/M phase depends on cell size and growth rate.

We model cell cycle dynamics using a G1 sizer model [47,48], where the cell cycle is split into two phases, G1 and S/G2/M (figure 1C). A newborn cell begins to grow in the G1 phase and remains there until its area exceeds a threshold value, $A_{S,\alpha }$, where α distinguishes the value for a given cell type. Once $A_{\alpha }\geq A_{S}$, a fixed countdown timer begins, which is the duration of the S/G2/M phase. After $\tau_{0}$ time has elapsed in the S/G2/M phase, the cell divides along the semi-major axis. Upon division, each daughter cell is assigned half the area of the mother cell, and its target area is initialized to match its actual area ($A_{\alpha }^{T}=A_{\alpha }$) to ensure mechanical equilibrium at birth. Both $\tau_{0}$ and $A_{S,\alpha }$ are drawn from Gaussian distributions that are derived from experimental data on Madin–Darby canine kidney (MDCK) cells [48]. Hence in our model, contact inhibition of proliferation results from tissue crowding halting cycle progression from G1 to S/G2/M [17].

We implement three different modes of cell elimination in our model, based on experimental observations: density-dependent apoptosis [28], contact-dependent apoptosis [12] and live-cell extrusion [18]. The initiation of density- and contact-dependent apoptosis are governed probabilistically by the local cellular environment, while extrusion operates deterministically, driven by the interplay between cellular and tissue-level states. We define the local density of a cell α as the sum of the inverse areas of the cells in direct physical contact with the cell α,


(2.4) $$\rho_{\alpha }=\frac{1}{A_{\alpha }}+\sum_{i\in \text{neighbours of }\alpha }\frac{1}{A_{i}}\;.$$

This definition of local density, as the sum of the inverse areas of neighbouring cells, was chosen to match the experimental analysis in Bove *et al.* [28] and more accurately captures local crowding and mechanical confinement than global density measures, such as (number of cells)/(total area covered by the cells), which can obscure spatial heterogeneity in mixed populations. The functional form for the dependence of the probability of apoptosis on local cell density is obtained by fitting a logistic curve to the experimental data of apoptosis probabilities [28] on MDCK cells (figure 1D),


(2.5) $$\begin{array}{lcr} & P_{\text{apo,den}}(\rho )=\frac{p_{\text{den,max}}}{1+e^{-\beta (\rho -\rho_{1/2})}}\;, & \end{array}$$

where $p_{\text{den,max}}$ is the maximum probability of apoptosis, $\rho_{1/2}$ is the cell density at half-maximum probability and β is the sensitivity to density-dependent apoptosis.

Similarly, the probability of contact-dependent apoptosis is obtained by fitting a Hill function to the data obtained by Levayer *et al.* [12] in *ex vivo Drosophila* wing discs (figure 1D),


(2.6) $$\begin{array}{lcr} & P_{\text{apo,con}}(p_{AB})=\frac{p_{\text{con,max}}p_{AB}^{n_{H}}}{S^{n_{H}}+p_{AB}^{n_{H}}}\;, & \end{array}$$

where $p_{AB}$ is the proportion of cell perimeter in heterotypic contact, $p_{\text{con,max}}$ is the maximum probability of apoptosis, S is the steepness parameter and $n_{H}$ is the Hill coefficient [35]. Contact-dependent apoptosis occurs specifically at heterotypic interfaces, where two genetically distinct cell types (Type A and Type B) are in contact. On initiation of apoptosis, regardless of the method, the cell’s target area is set to zero, which leads to a rapid decrease in area until eventually being removed from the tissue (see appendix B for details). Lastly, a cell α is eliminated via extrusion when its area is less than a quarter of its cell type’s mean area at that time step, $A_{\alpha }<\langle A\rangle_{\alpha }/4$. Upon meeting this condition, the cell is promptly deleted to mirror the short timescale of live cell extrusions, and its lattice sites are converted to medium lattice sites.

The apoptosis probability curves in our model are based on data from distinct biological systems: density-dependent apoptosis was fit to data from MDCK monolayers [28], where both crowding and contact effects may be present, while contact-dependent apoptosis was derived from *Drosophila* pupal notum data [12], where mechanical influences on apoptosis were not observed. These datasets were used due to the absence of comprehensive measurements within a single model system. To mitigate system-specific biases, we systematically varied model parameters to identify general principles governing competitive fitness.

Staking the physical and decision-making layers produces a predictive cell-autonomous model that can emulate the spatio-temporal tissue dynamics of cell competition. We now use this model to investigate the growth potential of a small colony of mutant cells surrounded by a tissue of wild-type cells. Unless otherwise stated, all simulations include both contact-dependent apoptosis and crowding-induced mechanical death mechanisms. The probability of contact-dependent apoptosis is identical for both cell types (unless otherwise stated), allowing us to isolate the effects of physical asymmetries.

## 3. Results

### 3.1. Differential compressibility drives cell competition

**Figure 2.**
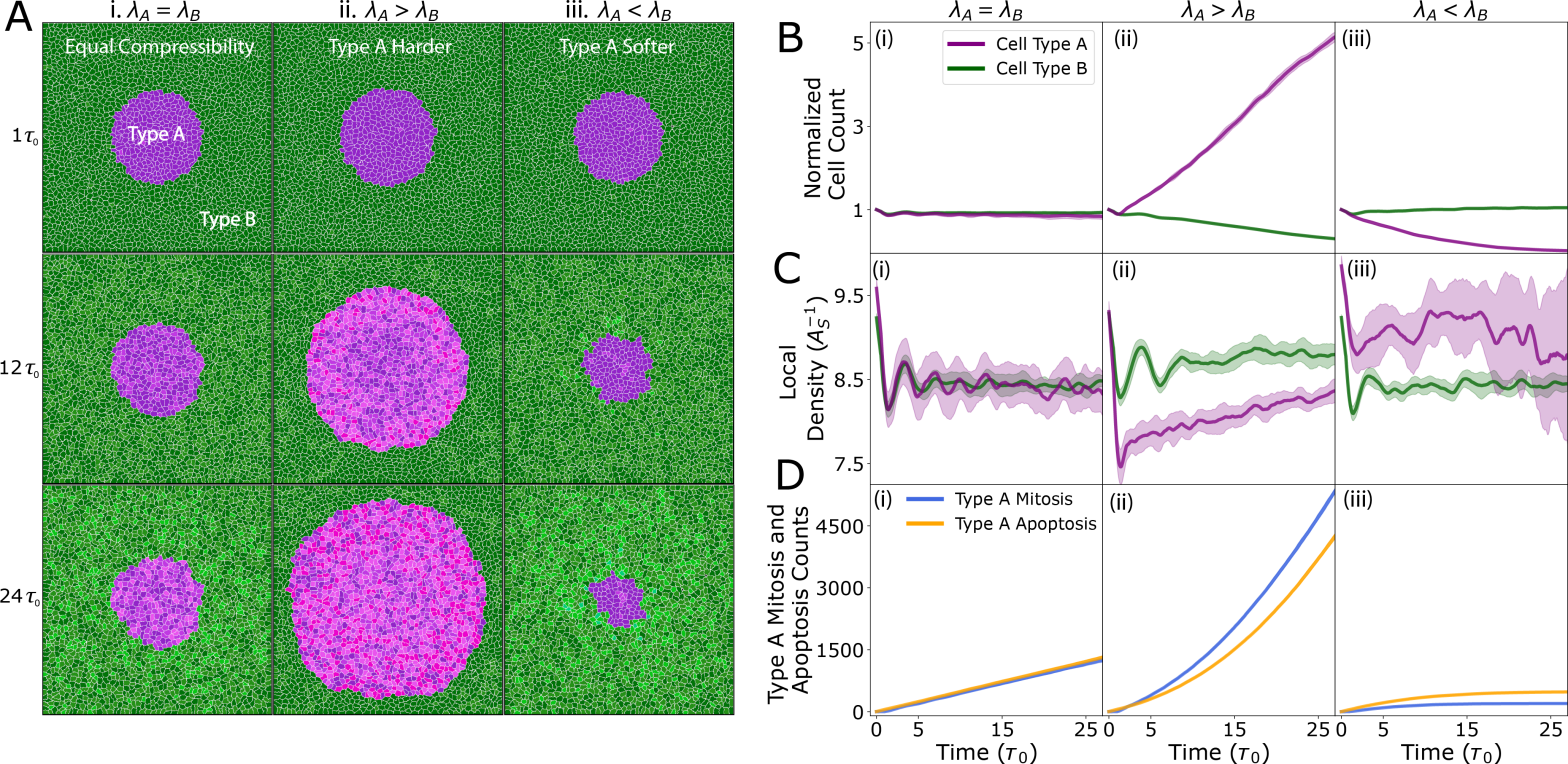
Differential compressibility alone drives cell competition. (A–D) Each column represents a different relative value of compressional elastic modulus of cell type A, $\lambda_{A}$, with respect to the compressional modulus of cell type B, $\lambda_{B}$ (with $\lambda_{B}=1$ for all simulations). All simulations start from the same initial configuration. Both contact-dependent apoptosis and crowding-induced cell death are active in these simulations. (A) Snapshots taken of simulations at $t=\tau_{0}$, 12$\tau_{0}$ and 24$\tau_{0}$ (first, second and third rows, respectively). Columns indicate the compressional elastic modulus of type A cells when compared with type B cells: (i) $\lambda_{A}=\lambda_{B}$, (ii) $\lambda_{A}>\lambda_{B}$, (iii) $\lambda_{A}<\lambda_{B}$. (B–D). The plots show the (*n* = 5) average value (darker line) and standard deviation (lighter colour). (B) Normalized cell count (N(t)/N(0)) as a function of time (purple for type A and green for type B). The more compressible cells are eliminated from the tissue while the equally compressible cells survive. (C) Local density as a function of time (purple for type A and green for type B). Cells with a lower relative elastic modulus are compressed above their homeostatic density, which increases their probability of mechanical apoptosis. (D) Counts of mitosis and apoptosis for type A cells. (iii) With $\lambda_{A}<\lambda_{B}$, type A cells are eliminated, in part, due to their low division count, which indicates an inability to grow. See tables 1 and 2 for a list of default model parameters. The cells change colour upon division to make these events easier to see. Both cell types cycle through four colours. Type A generations: 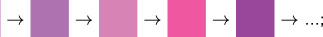 Type B generations:
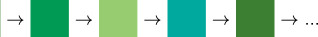

A viable cell must sustain adequate rigidity to facilitate growth and division. Prior investigations have shown that MDCK cells depleted of the polarity gene *scribble* are eliminated when surrounded by wild-type cells, a phenomenon attributed to mechanical competition [28]. Although there has been no direct measurement of stiffness of *scribble* knockdown cells, model-based inference suggests that they have a lower rigidity than wild-type cells [35]. Furthermore, differential cell stiffness is also associated with mechanical competition between normal and bacterially infected cells [49], and also in cancer cell invasion [50–53]. While our model does not explicitly map to these specific biological systems, we investigated how differences in cell compressibility alone could lead to fitness differences in the cell population. To this end, we simulated the growth of a small colony of Type A cells with a different compressibility compared with the surrounding Type B cells (figure 2A, electronic supplementary material, videos S1–S3). Simulations were performed in a fixed 1500 × 1500 pixel domain with non-periodic boundaries. At initialization (t=0), all cells are assigned area equal to their preferred resting area ($A=A_{0}$), ensuring that the tissue is mechanically relaxed at the outset. To isolate the effects of compressibility, all other cellular parameters for types A and B were kept fixed at their default values. See tables 1 and 2 and appendix C for details on parameter choices.

**Table 1.** Default parameter values used in model simulations.

parameter	cell types A and B	parameter description
λ	1	cell area elastic modulus
G	1.14 ( $A_{S_{0}}$ / $\tau_{0}$ )	growth rate of an isolated cell
k	1.96 × $10^{5}$ ( $A_{S}^{-2}$ )	sensitivity to crowding
$\tau_{0}$	225 ± 5.625 (Monte Carlo steps)	S/G2/M timer
$A_{S}$	1400 ± 35 (pixels)	G1 sizer threshold
$p_{\text{den,max}}$	0.0015	maximum probability of apoptosis
$\rho_{1/2}$	14.01 ( $A_{S}^{-1}$ )	cell density at half-maximum probability of apoptosis
β	0.104 ( $A_{S}$ )	sensitivity to density-dependent apoptosis
$p_{\text{con,max}}$	1.0	maximum probability of contact-dependent apoptosis
S	1.35	contact perimeter proportion at maximum probability
$n_{H}$	3.6	sensitivity to contact-dependent apoptosis
$k_{B}T$	10	thermal energy in CPM

**Table 2.** Default contact energies used in model simulations. All energy values are expressed in units of the characteristic energy scale $\lambda A_{S}^{3/2}$ .

contact energies	X = type A	X = type B
$J_{\text{XX}}$ (homotypic)	2.39 × $10^{-4}$	1.15 × $10^{-4}$
$J_{\text{XY}}$ (heterotypic)	1.91 × $10^{-4}$	1.91 × $10^{-4}$
$J_{\text{XM}}$ (cell-medium)	2.86 × $10^{-4}$	2.86 × $10^{-4}$

When the system is composed of cells of identical compressional modulus ($\lambda_{A}=\lambda_{B}$), the population dynamics of type A and type B cells are fairly identical across time (figure 2Bi, electronic supplementary material, video S1), the average local densities are approximately similar (figure 2Ci), and the rates of mitosis and apoptosis of the type A colony are nearly equal over time (figure 2Di). The simulation snapshots in figure 2Ai show minor morphological changes in colony A, but qualitatively, colony A size is unchanged over a long timescales. Type A cells with the same stiffness and adhesion parameters as type B cells serve as a model for a pseudo-clone—i.e. a tracked subset within an otherwise homogeneous population. Simulations of such a pseudo-clone (electronic supplementary material, figure S1) exhibit no competitive advantage, confirming that our framework yields neutral dynamics under homotypic conditions, as expected for pseudoclones.

By contrast, simulations with softer type B cells ($\lambda_{A}>\lambda_{B}$), where *softer* refers to a lower area elastic modulus λ (i.e. greater compressibility), which causes the cells to be more easily deformed under pressure, are subsequently eliminated via mechanical competition (figure 2Aii,Bii, electronic supplementary material, video S2). Consequently, we observe a higher rate of mitosis in Type A cells compared with the rate of apoptosis (figure 2Dii). In the case of softer type A cells $\lambda_{A}<\lambda_{B}$ (figure 2Aiii, electronic supplementary material, video S3), they are compressed beyond their equilibrium density (figure 2C, compare purple curves in panels i and iii) and are then eliminated preferentially (figure 2Biii). This results from a higher rate of apoptosis of Type A cells compared with the rate of mitosis. These data collectively indicate that cell compressibility is a key factor in determining the viable mechanical pressure at which growth and proliferation occur, and when a cell’s environmental pressure exceeds this viable pressure, then growth effectively halts and the cell cycle is arrested in the G1-sizer phase. Since all other parameters are being held equal in these simulations, the higher density of cells with lower compressional modulus represents a decrease in fitness due to it also lowering the rate of division and increasing the rate of elimination: small cells cannot proliferate due to halting in the G1-sizer phase, and cells with a high local density have a greater probability of death (equation (2.5)).

### 3.2. Trade-off between cell compressibility and sensitivity to crowding controls the outcome of cell competition

Our simulations thus far indicate that cells with a higher compressional elastic modulus emerge as supercompetitors, when all other cellular properties are identical. This finding contrasts with expectations for cancer cells, which tend to be softer than normal cells [51–53] but still outcompete them. We then explored the extent to which a reduction in fitness due to a lower compressional modulus could be counterbalanced by an increase in fitness driven by another parameter. Since cancer cells are characterized by a lower contact inhibition of proliferation [16,54,55], we varied the ratio of the sensitivity to crowding, $k_{A}/k_{B}$, along with the ratio of compressional moduli $\lambda_{A}/\lambda_{B}$, in order to investigate their combined effects on the fitness of the mutant colony A.

**Figure 3.**
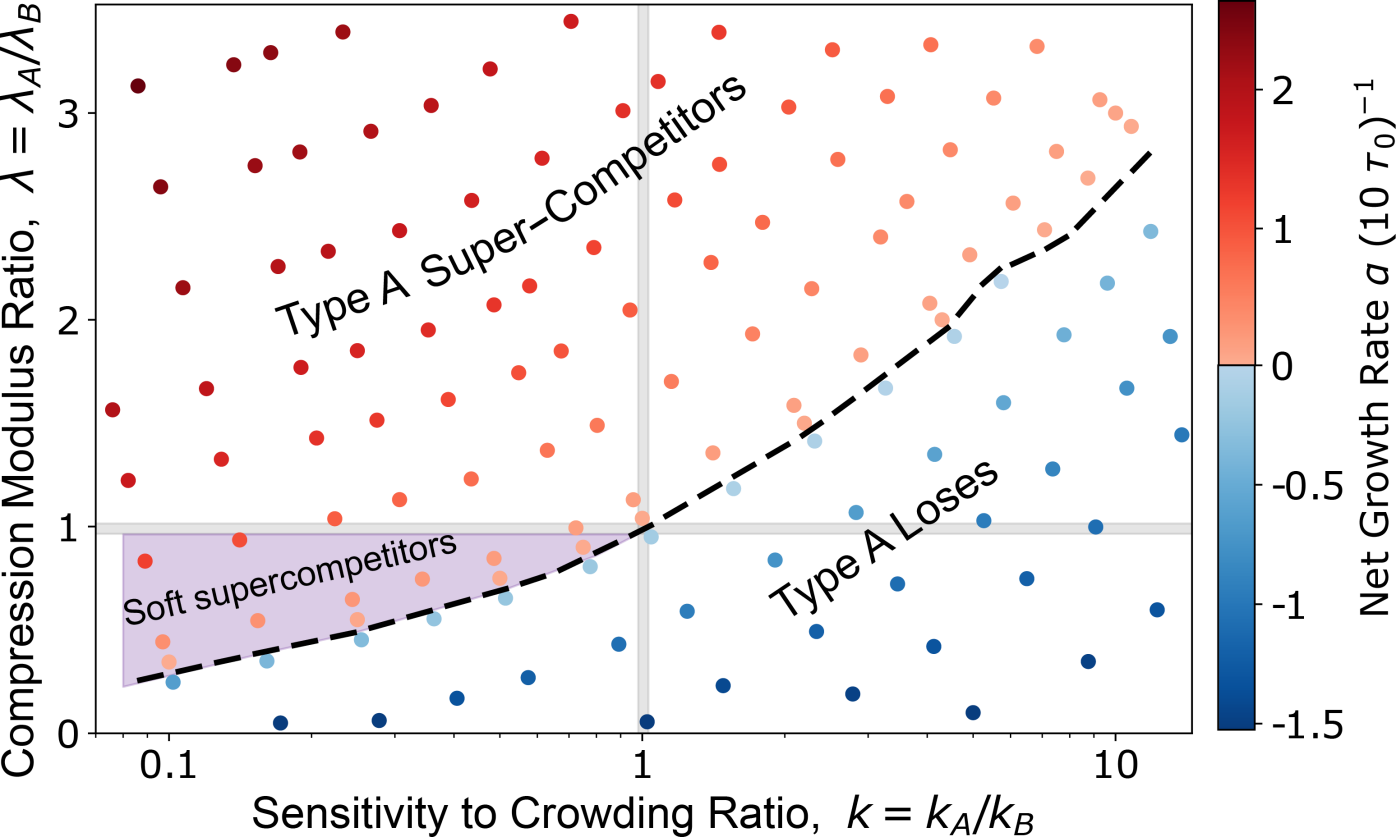
Trade-off between compressional elastic modulus and sensitivity to crowding determines outcome of cell competition. Phase diagram showing the net growth rate, α, of type A cells over the phase space of compressional elastic modulus ratio, $\lambda =\lambda_{A}/\lambda_{B}$, and the sensitivity to crowding ratio, $k=k_{A}/k_{B}$. The dashed black line represents where the net growth is approximately zero. Above this line type A is supercompetitive, and below the line type A loses to type B. The purple region (λ<1, k<1, and above the dashed line) emphasizes where type A cells are more fit despite being softer than their type B neighbours. See tables 1 and 2 for a list of default model parameters.

To characterize the fitness of colony A, we measured the net growth rate α, defined as $\alpha =N^{-1}\text{d}N/\text{d}t$, where N(t) is the cell count of type A cells at time t. We determined α by fitting an exponential function to the average (n=6) population size (figure 3). By varying $\lambda_{A}/\lambda_{B}$ and $k_{A}/k_{B}$, we mapped the fitness landscape (figure 3), identifying regions where type A tissues exhibit supercompetitive behaviour (red points, α>0), where type A tissues are eliminated (blue points, α<0), and where type A and B tissues coexist in an unstable equilibrium (on dashed line, α≈0). Corresponding phase diagrams of division rates and death rates are shown in electronic supplementary material, figure S2. As we discussed in the previous section, an increase in compressional elastic modulus is associated with an increase in cellular fitness. However, we find that the decrease in fitness from softer (more compressible) cells can be compensated for by a proportional reduction in sensitivity to crowding, such that there is a net increase in fitness (figure 3, purple shading). We thus identify a region in λ-k phase space that correspond to soft supercompetitors, where softer type A cells are able to outcompete harder type B cells due to their lower sensitivity to crowding. This finding is consistent with observations that many cancerous cell lines are typically softer than normal cell lines [53,56]. While we do not model specific cancer genotypes, our results suggest that such physical traits—when combined with reduced crowding sensitivity—may contribute to competitive expansion. We further verified that this phase diagram remains robust to variations in homotypic adhesion strength (i.e. reversing the relationship between $J_{AA}$ and $J_{BB}$) under the conditions studied (electronic supplementary material, figure S3).

### 3.3. Mutant fitness is governed by physical parameters that regulate relative growth potential and susceptibility to apoptosis

**Figure 4.**
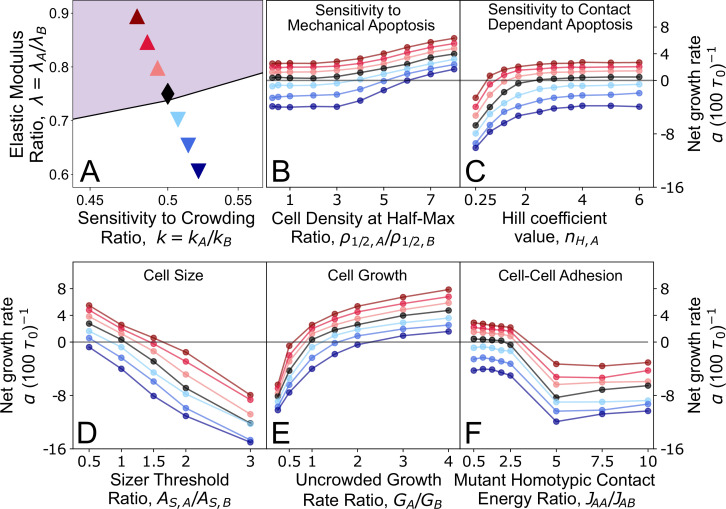
Mutant colony fitness is primarily determined by relative growth potential and susceptibility to apoptosis. (A) Points on the (λ,k) phase space, representing type A colonies with different values of elastic modulus ratio λ, and sensitivity to crowding ratio, k. Out of these seven representative cell colonies, three are supercompetitors (α>0, purple shading), three are losers (α<0, white shading) and one lies on the line of coexistence (black point). (B) Net growth rate of type A colonies as a function of $\rho_{1/2,A}/\rho_{1/2,B}$, the ratio of the cell densities at half-maximum apoptosis probability (equation (2.5)). Changing $\rho_{1/2,A}$ alters the sensitivity to mechanical apoptosis of type A cells. (C) Net growth rate of type A colonies as a function of $n_{H,A}$, the Hill coefficient in the equation for the probability of contact-dependent apoptosis (equation (2.6)). Changing $n_{H,A}$ alters the sensitivity to contact-dependent apoptosis of type A cells. Dashed line indicates the critical value $n_{H,A}=3.6$ above which the probability of apoptosis decreases with increasing $n_{H,A}$. (D) Net growth rate of type A colonies as a function of the ratio of sizer threshold for type A and B cells. Cells that grow larger to progress through their cell cycle are less fit and more susceptible to apoptosis. (E) Net growth rate of type A colonies as a function of the ratio of uncrowded growth rate for type A and B cells. Cells that grow faster are more fit and emerge as supercompetitors. (F) Net growth rate of type A colonies as a function of the ratio of type A homotypic contact energy, $J_{AA}$, over heterotypic contact energy of type A and B cells, $J_{AB}$. For $J_{AA}/J_{AB}>2.5$, there is a substantial increase in heterotypic contact, leading to an increase in susceptibility of type A cells to contact-based apoptosis.

Having identified the parameter regimes in λ-k phase space where the mutant type A cells emerge as supercompetitors (figure 3), we sought to investigate how perturbations in other physical cell parameters impact the fitness landscape. To understand how the fitness landscape transforms under parameter perturbations, we investigated seven representative type A colonies (see figure 4A) that traverse the border of supercompetition and elimination. We then varied the model parameters that regulate cell proliferation and death dynamics for type A cells, including the cell growth rate, strength of homotypic adhesion relative to heterotypic adhesion, the G1 sizer threshold, sensitivities to density-dependent apoptosis and contact-dependent apoptosis.

#### 3.3.1. Sensitivity to mechanically driven apoptosis

Differential sensitivity to mechanical pressure has been identified as a key factor in the elimination of cells through mechanical competition [8]. Cells with higher growth and proliferation rates exert pressure on their slow-growing neighbours, leading to an accumulation of mechanical stress [7]. If slower-growing cells are more sensitive to pressure or density-dependent apoptosis, they are more likely to be eliminated. For instance, in MDCK monolayers, *scribble* mutant cells are out-competed due to their heightened sensitivity to mechanical compaction [9,28]. While our model does not simulate the full phenotype of *scribble* mutants, we use these observations to motivate parameter perturbations in cell death sensitivity.

To test the effects of alterations in mechanically driven cell elimination, we altered type A cell’s sensitivity to density-dependent apoptosis by varying the density $\rho_{1/2,A}$ at half-maximum probability of apoptosis (equation (2.5)). This means that if $\rho_{1/2,A}>\rho_{1/2,B}$, then type A cells have a lower probability of death for any given value of local density. A sufficiently large difference in $\rho_{1/2}$ values can effectively switch off density-dependent apoptosis for type A cells. We found substantial increases in the net growth rate of type A cells at higher values of the ratio $\rho_{1/2,A}/\rho_{1/2,B}$ (figure 4B). For ratios $\rho_{1/2,A}/\rho_{1/2,B}\geq 4$, type A cells became supercompetitors, preferentially eliminating type B cells. By contrast, lower values of $\rho_{1/2,A}/\rho_{1/2,B}$, which effectively sets $P_{\text{apo,den}}\approx p_{\text{den,max}}$, had minimal effect on the net growth rate of type A cells.

#### 3.3.2. Sensitivity to contact-based competition

Prior experiments on *myc*-driven competition in the *Drosophila* pupal notum have shown that cells expressing higher levels of *myc* preferentially eliminate wild-type cells when they are in mutual contact [12]. In quantitative terms, the probability of apoptosis for wild-type cells is proportional to the amount of heterotypic shared contact (figure 1D-bottom). In our simulations, we altered the sensitivity to contact-dependent apoptosis by adjusting the Hill coefficient $n_{H}$ in the probability (equation (2.6)): $P_{\text{apo,con}}(p_{AB})=p_{\text{con,max}}p_{AB}^{n_{H}}/(S^{n_{H}}+p_{AB}^{n_{H}})$, where the parameters $p_{\text{con,max}}$ and S are obtained by fitting data [12] (figure 1Dii). For a fixed value of S, increasing the Hill coefficient $n_{H}$ steepens the transition in the apoptosis probability: it lowers the probability for cells with $p_{AB}<S$ and raises it for those with $p_{AB}>S$, without affecting the value at $p_{AB}=S$. Thus, by changing the Hill coefficient for type A cells, $n_{H,A}$, the probability of apoptosis can be increased from its default value for $n_{H,A}<3.6$ or decreased for $n_{H,A}>3.6$ (figure 4C). Interestingly, greatly decreasing the probability of apoptosis, by increasing $n_{H,A}$, resulted in a negligible change in colony A fitness. This is consistent with our prior study which showed contact-dependent apoptosis dominated the outcome of competition only when the loser cells were fully mixed with the winner cells [35]. As expected, increasing the probability of contact-dependent apoptosis, by decreasing $n_{H,A}$, gradually decreases the net growth rate of all tissues.

#### 3.3.3. Perturbations to cell size and growth

Mutations in ribosomal proteins are one of the main drivers of cell competition [5,57]. Reduced translation and slow growth affects cell size, suggesting that differential cell size and growth rate may be sensing cues for cell competition. We therefore asked how perturbations to cell size determine the emergence of supercompetitors. In our simulations, cell size is controlled by the G1/S sizer threshold, $A_{S}$. The sizer threshold determines the minimum size necessary for a cell to progress from the G1 phase to the S/G2/M phase. In a crowded tissue, where growth is slow and dependent on the apoptosis of nearby cells to free space, the average cell cycle time is dominated by the duration of the G1 phase (figure 1C). Alterations to cell size can tilt the balance between cell proliferation and apoptosis rates in two ways. First, a smaller $A_{S}$ will reduce cell cycle times, thereby increasing proliferation rates. Secondly, larger cells will typically have more neighbours, hence a higher local density and a greater probability to die via mechanical and contact-based competition. We thus expect cell fitness to decrease with increasing cell size. In confirmation of this, we found an inverse linear relationship between the population net growth rate and sizer threshold for all type A colonies (figure 4D). The increase in fitness for the smaller sizer threshold is primarily due to the shorter cell cycle times, representing an overall increase in proliferation rate relative to the rate of apoptosis. Consistent with this finding, *scribble* mutants that acquire a loser phenotype in competition with MDCK wild-type cells, are typically larger in size [28,35].

It has been suggested that differential growth rate can lead to the accumulation of mechanical stresses at the interface between two competing cell types [7,23], leading to the elimination of the slower-growing cells via mechanically driven apoptosis. To test this in our model, we varied the uncrowded growth rate G of type A cells (equation (2.3)), the maximum growth rate in the absence of crowding. We found that altering the uncrowded growth rate of type A cells, $G_{A}$, significantly impacted tissue fitness (figure 4E), making type A cells either supercompetitors for higher growth rates or losers for lower growth rates. From this result, in conjunction with figure 3 and electronic supplementary material, figure S4, we find that the tissue growth potential, regulated by the parameters λ, k and G, is crucial for determining the outcome of cell competition.

#### 3.3.4. Cell–cell adhesion

Loss of cell–cell adhesions is a key feature of the epithelial–mesenchymal transition, during which epithelial cells acquire migratory and invasive characteristics [58]. While increased motility in supercompetitors can trigger apoptosis of suboptimal cells through contact-based elimination, weakened homotypic adhesions can also affect the mechanical properties of the mutant colonies, such as their compressibility. A recent study suggests that reduced cell–cell adhesions in HRas${,}^{\text{V12}}$-expressing transformed cells contribute to their elimination by wild-type cells due to increased compressibility of the transformed cells [38].

To explore the role of intercellular adhesions in cell competition through our simulations, we varied the ratio of homotypic contact energy ($J_{AA}$) to heterotypic contact energy ($J_{AB}$), while keeping the other parameters fixed. Since a lower contact energy implies stronger adhesion, increasing $J_{AA}/J_{AB}$ weakens homotypic adhesion relative to heterotypic adhesion, thereby promoting greater intermixing between cell types A and B. We observed that for $0.5<J_{AA}/J_{AB}<2.5$, there is minimal effect on the net growth rate of colony A, despite incremental increases in heterotypic contact. However, when $J_{AA}/J_{AB}$ exceeds 2.5, a significant drop in net growth rate occurs as type A cells begin to repel one another, leading to type A cells migrating into the type B tissue and creating a fully mixed state (electronic supplementary material, video S4). In this state, heterotypic contact—and consequently, the probability of contact-dependent apoptosis—is maximized, resulting in the complete elimination of type A cells. Therefore, increasing homotypic contact energy (and thereby weakening homotypic adhesions) leads to the elimination of mutant cells at sufficiently high $J_{AA}/J_{AB}$ values. We note that homotypic contact energy of the wildtype cells, $J_{BB}$, has little to no effect on the dependence of α on $J_{AA}/J_{AB}$ (see electronic supplementary material, figure S5).

### 3.4. Critical size for colony growth and survival

The size of mutant colonies plays a crucial role in determining their growth, survival and invasive potential. Previous theoretical studies have suggested a critical size threshold for the elimination of mutant colonies [15,59–61], consistent with experiments on the influence of colony size on tumour invasion and cell removal [62–64]. However, multiple factors contribute to whether a colony is eliminated by the surrounding tissue. Mechanistically, smaller colonies would experience a higher Laplace pressure (due to their higher interface curvature) compared with larger colonies, which may lead to their elimination through the compressive Laplace pressure [63]. This effect depends on the rigidity of the mutant colony compared with the surrounding tissue, as well as the homotypic contact energy that controls the colony surface tension. In addition, smaller colonies, with a higher perimeter-to-area ratio, are more susceptible to elimination through contact-based competition due to increased heterotypic contact at the boundary. Therefore, mechanical and contact-based modes of cell elimination may either act antagonistically or act in synergy with each other depending on the geometry and mechanical properties of the tissue [65].

**Figure 5.**
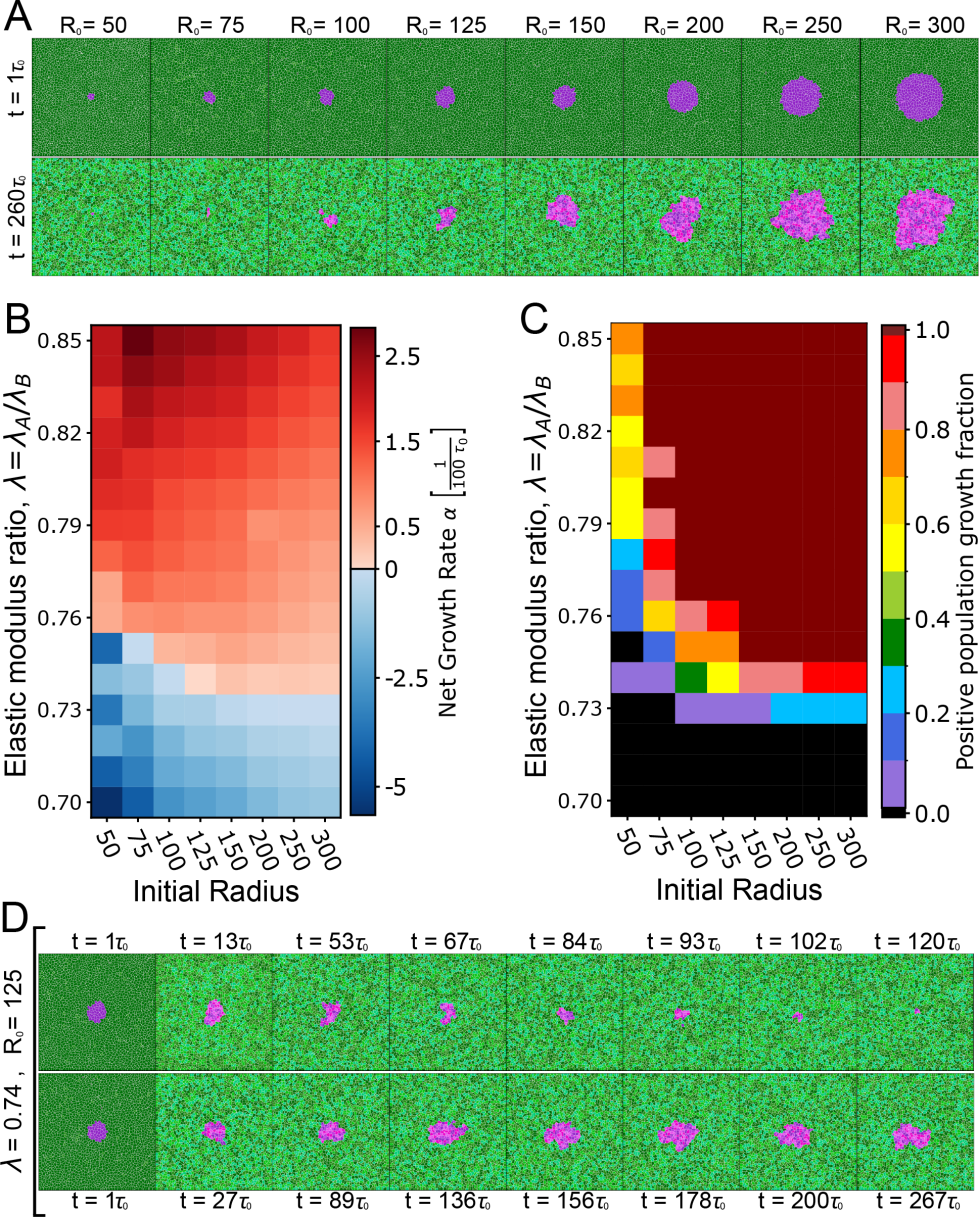
Critical colony size depends on relative cell compressibility. (A) Top row: Snapshots near the start of the simulation ($t=\tau_{0}$) depicting the initial size of colony A in terms of the radius (in pixels). Bottom row: Configuration of the system at $t=260\tau_{0}$ showing either extinction or survival. (B) Colourmap of net growth rate of colony A, as a function of different initial sizes and ratio of compressional moduli. The sensitivity to crowding ratio is set to k=0.5, such that type A cells are less sensitive to crowding-induced suppression of growth. Each bin represents an average over 30 simulations. The net growth rate changes from negative to positive with increasing initial radius for $\lambda_{A}/\lambda_{B}$ in the range [0.74,0.75]. (C) Phase diagram (colourmap) showing the fraction of simulations which had positive population growth rate at $t=260\tau_{0}$; that is $N_{A}(260\tau_{0})>N_{A}(0)$ where $N_{A}(t)$ is the population of the A colony at time t. (D) Time-evolution of a colony of initial size R=125 pixels at λ=0.74, showing an instance of elimination (top row) and survival (bottom row).

To understand how the interplay between colony size and mechanical properties impact their growth and survival, we varied the initial size of colony type A in our simulations across different values of the relative compressibility $\lambda =\lambda_{A}/\lambda_{B}$. We held the sensitivity to crowding ratio constant $k=k_{A}/k_{B}=0.5$, assuming that type A cells have a lower sensitivity to crowding, mimicking cancer cell colonies. The initial configuration consisted of a colony of type A cells of a specified radius (*R* = 50–300 pixels), positioned at the centre and surrounded by type B cells (figure 5A). We found that for a given value of relative compressibility λ (e.g. λ=0.74 in figure 5A), there is a critical colony size R∼125 pixels, below which the colony is eliminated, while above it, the colony continues to grow and survive (electronic supplementary material, videos S5–S7). Here contact-based competition enabled type B tissue to eliminate the otherwise supercompetitive type A colonies (for λ=0.74, k=0.5, see figure 3), particularly when type A colonies were below a critical size. Thus, different initial sizes correspond to different sensitivities to contact-based elimination.

**Figure 6.**
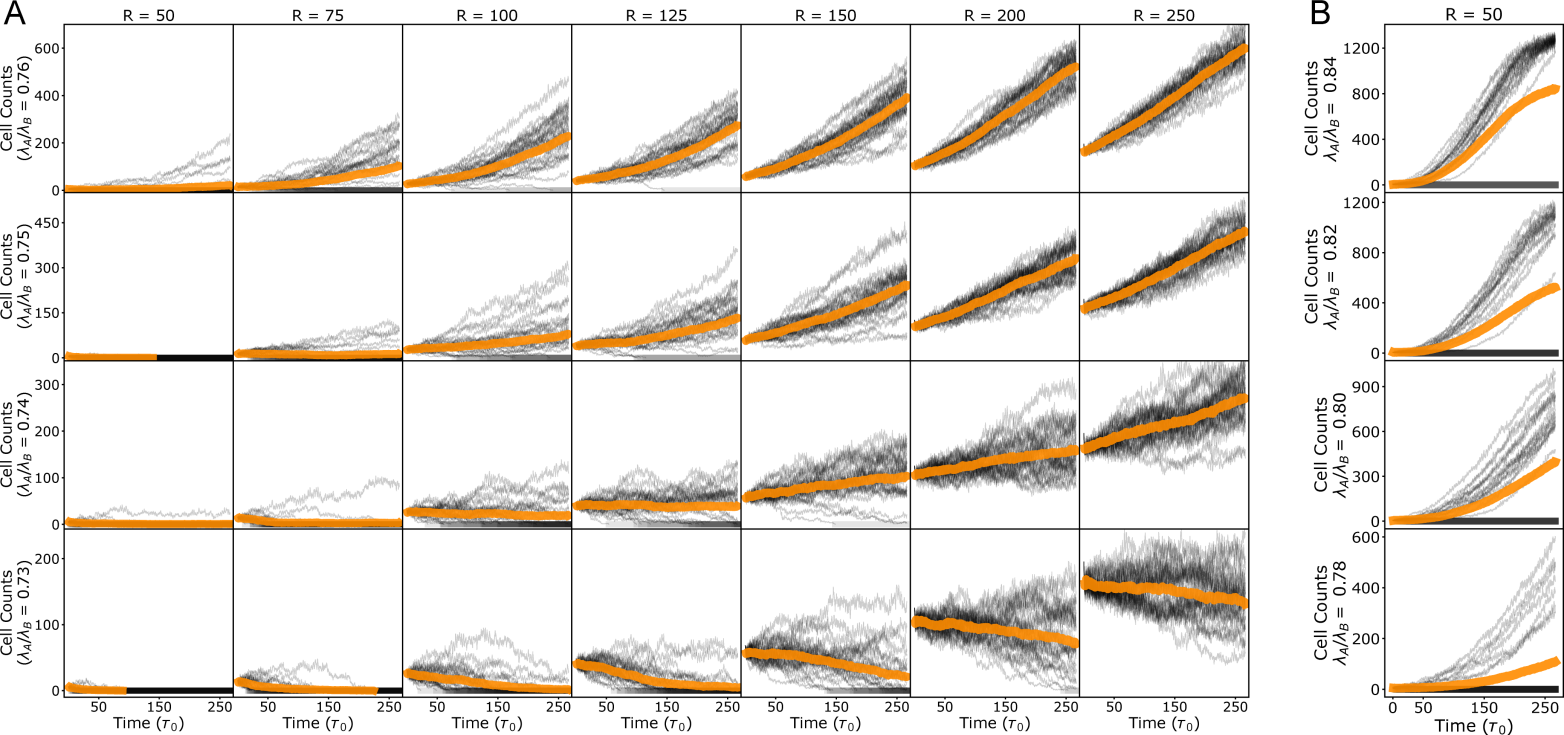
Population growth trajectories for varying initial sizes and relative compressibility. (A,B) Population growth trajectories (cell count versus time) for type A colonies from individual simulations (black lines) and their average population (n=30, orange lines). The parameters for type A colonies are indicated in each row with the relative compressibility $\lambda =\lambda_{A}/\lambda_{B}$ and by each column with the initial radius in pixel units. The sensitivity to crowding ratio is kept fixed at $k_{A}/k_{B}=0.5$. (A) Despite type A cells possessing a greater relative growth potential, for λ≥0.74 and k=0.5, smaller type A colonies can be outcompeted by the otherwise less fit type B tissue. This is evidenced by a decline in cell count in some simulations. For instance, note the comparisons: λ=0.74 for R=100→150, λ=0.75 for R=75→100 and λ=0.76 for R=50→75. (B) Population growth trajectories for initial size R=50 pixels at different values of relative compressibility. Type B tissues can outcompete more fit type A colonies (see leftmost column of panel A), though with reduced frequency proportional to their relative compressibility.

The critical colony size depends on the relative compressibility, λ, such that for λ=0.75 cells exhibited a strictly positive net growth rate when the initial size R>75 pixels (figure 5B). For λ≤0.73, the average (n=30) net growth rate is negative, implying that type A colonies would be eliminated regardless of their initial size (figure 5B). By contrast, for λ≥0.76, the average net growth rate is positive, and type A colony invades by eliminating the type B cells. However, this behaviour is probabilistic (figure 5C), such that for λ>0.73 there exists a compressibility-dependent critical size below which several simulations showed an increase in the population count of type A cells (figure 6A). For the smallest colony size (R=50 pixels, consisting of six type A cells), type B tissue managed to eliminate type A cells in some simulations, despite type A being more fit (λ≥0.76, figures 5C and 6B). Together, these results show that colony size impacts its fitness, but the critical colony size for growth is dependent on tissue mechanical properties and is governed by a probabilistic process.

**Figure 7.**
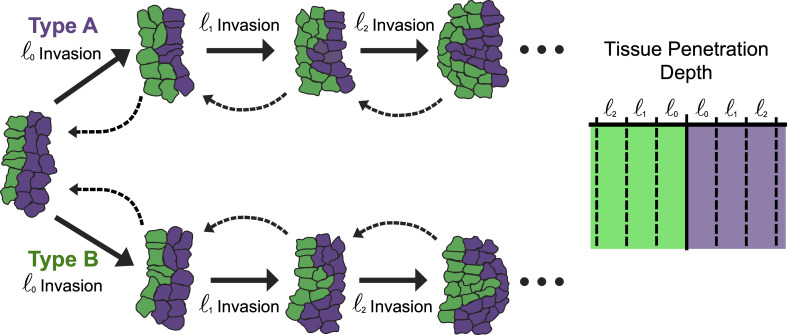
Colony invasion mechanism. Schematic showing the stochastic nature of invasion of a cell type onto another, initiated by a finger-like instability at the boundary between the two cell types. Invasions increase heterotypic contact yielding an average decrease in fitness for the small type A colonies due to a lack of replacement bulk cells. The death of a boundary cell allows for the initial invasion of the opposite type by an amount less than the typical cell size. This tends to increase the proportion of heterotypic shared contact, especially for each invading cell and the boundary cells neighbouring the invasion. The invasion then progresses or is reversed in a probabilistic process that is most dependent on the ability of the tissue bulk cells to grow and replace the apoptotic boundary cells.

The stochastic nature of cell colony elimination is illustrated in figure 5D, where we see that two type A cell colonies of the same initial size and identical physical properties show different outcomes in different simulation runs—one in which the colony survives (figure 5D, bottom row) and the other where the colony is eliminated (figure 5D, top row). The mechanism for elimination or invasion can be explained as follows (see figure 7). When a cell at the boundary between two cell types dies, it permits a local invasion by the opposite type, with the extent of the invasion being slightly smaller than the typical cell size. This invasion increases the proportion of heterotypic contact, particularly for the invading cells and the boundary cells adjacent to the invasion site. This increase in heterotypic contact would increase the probability of contact-dependent apoptosis, promoting further invasion. However, the invasion can be reversed if the opposite type’s tissue cells proliferate and replace the apoptotic boundary cells. Thus, a competition between proliferation and apoptosis of boundary cells determines whether the invasion will progress or be reversed.

## 4. Discussion

In this study, we developed a cell-based model of cell competition that integrates cellular mechanics and decision-making rules using the framework of the cellular Potts model (figure 1). The model allows for control over individual cell properties—such as mechanical forces, cell cycle parameters and apoptosis rules—enabling us to identify the physical traits of individual cells that determine their potential to become supercompetitors. We explored this by embedding a mutant clone within a confluent tissue with distinct physical and biochemical properties, a situation relevant to developmental processes or early tumour formation. Notably, our results show that populations with similar apoptosis profiles may experience divergent outcomes due to differences in proliferation rate or crowding sensitivity, emphasizing that growth dynamics are essential to understanding competitive fitness.

We demonstrate that differential compressibility alone can drive competition between otherwise identical cell types (figure 2). Specifically, cells that are stiffer and more resistant to compression emerge as supercompetitors. This occurs because the stiffer cells compact the softer ones, increasing the local density of the softer cell type, making them more susceptible to elimination through density-dependent apoptosis. However, when cell types differ in their sensitivity to crowding or contact inhibition of proliferation, the outcome of competition is governed by a balance between compressibility and sensitivity to crowding. For example, increasing the sensitive to crowding parameter of the mutant cells generally leads to their elimination, even when they are stiffer than the wild-type cells. Interestingly, we identified a region in parameter space where softer mutant cells can outcompete wild-type cells that have a lower sensitivity to crowding (figure 3). This finding is particularly relevant to cancer, as many cancerous cell lines are softer than their wild-type counterparts [66] and exhibit lower contact inhibition of proliferation [67]. Indeed, prior studies have shown that decreased cell stiffness correlates with increased malignancy in various cancers, including colon, ovarian and breast cancers [51,68–71].

The emergence of soft supercompetitors with low crowding sensitivity can also be influenced by other single-cell parameters, such as those regulating proliferative fitness or susceptibility to apoptosis (figure 4). For example, reducing the sensitivity to density-dependent or contact-dependent apoptosis can promote supercompetitive behaviour in parameter regimes where mutant colonies would otherwise be driven to extinction. Similarly, enhancing proliferative fitness by increasing growth rates or reducing the G1 size threshold can also induce supercompetitive behaviour. Another key physical factor is the ratio of homotypic to heterotypic contact energy ($J_{AA}/J_{AB}$), which reflects the relative adhesion between cell types A and B compared with adhesion within type A cells. Higher $J_{AA}/J_{AB}$ values promote greater intermixing of cell types, increasing heterotypic contact and thus making type A cells more vulnerable to elimination through contact-dependent apoptosis. This aligns with experimental evidence showing that increased cohesion within loser cells protects them from elimination, whereas greater intermixing between winner and loser cells leads to enhanced elimination of the losers [12].

The initial size of a mutant colony plays a crucial role in its survival probability, depending on its compressibility (figure 5). If the mutant cells are softer, colony growth is inhibited regardless of initial size. However, when compressibility exceeds a critical threshold, there is a critical initial size above which the colony is more likely to invade. Below this critical size, colony survival is stochastic, driven by a finger-like instability at the boundary between cell types (figure 7). Local apoptosis in the mutant clone creates space for wild-type cell proliferation, promoting invasion into the mutant colony. This invasion increases heterotypic contact, which in turn drives further apoptosis and invasion. Interestingly, our model predicts that this invasion can be reversed if the mutant clone has a higher compressional modulus or lower contact inhibition of proliferation, highlighting the importance of physical cell parameters in determining supercompetitive behaviour.

Our modelling strategy isolates the effects of individual cellular parameters—such as stiffness, crowding sensitivity or apoptosis regulation—rather than modelling specific genotypes that co-modulate multiple traits. This complements previous work on *scribble* mutants [35], where decreased proliferation and increased apoptosis sensitivity were modelled together. Here, by modulating one property at a time, we uncover how each trait can independently promote or suppress competitive fitness. This reductionist approach helps reveal biophysical design principles and may guide future experiments aimed at dissecting causality in cell competition.

While our model governs crowding-induced apoptosis using a local density metric—defined as the inverse of the average apical area of a cell and its neighbours—recent experimental work suggests that single-cell apical area may be a more direct physiological cue for cell fate decisions [72]. Although both formulations predict higher death rates for smaller cells, area-based rules may avoid certain paradoxical scenarios and offer greater biological realism. Importantly, our main conclusions remain robust across a range of parameters, and our simulations reproduce the experimentally observed trend that smaller cells are more susceptible to elimination.

## Data Availability

This article has no additional data. Simulations codes are available at Zenodo [73]. Supplementary material is available online [74].

## Appendix A. Cellular Potts model simulation

We utilized the cellular Potts model (CPM) [42] to simulate the dynamics of cell competition. The CPM is a versatile computational framework widely used to model biological processes such as cell growth, migration and tissue morphogenesis. The model operates by running a Monte Carlo simulation using the Metropolis–Hastings algorithm [45,46], which minimizes the system’s free energy, as described in the main text. While alternative cell-based modelling approaches exist, including vertex models, cell-centre models and particle-based models, we selected the CPM for its simplicity in integrating key components of this study, such as agent-based rules, boundary conditions, and cell-extracellular medium interactions. The simulations were conducted using CompuCell3D [43], a software package that implements the CPM framework and the Metropolis–Hastings algorithm. The version of CompuCell3D used in this study allows for cell fragmentation based on lattice temperature, which we set to $k_{B}T=10$ in lattice energy units. This temperature was found to result in negligible fragmentation. Simulations are initiated from confluent type B tissue in homeostasis on a two-dimensional lattice of 1500 × 1500 pixels. At time t=0, cells within a radius of 300 pixels (default value for initial radius) of the centre of the tissue are transformed to type A cells (initial population counts are $N_{A}$= 284 cells and $N_{B}$= 1931 cells). Other default parameters are specified in tables 1 and 2. Custom simulation scripts are available at Zenodo [73].

## Appendix B. Rules for cell elimination

In our simulations, cell elimination occurs via two distinct mechanisms: programmed cell death (apoptosis) and live-cell extrusion. Although the downstream apoptotic machinery may overlap, our model differentiates the initiation contexts for cell elimination: (i) density-dependent apoptosis triggered by mechanical compression and high local density; (ii) contact-dependent apoptosis triggered by extensive heterotypic interface; and (iii) extrusion triggered by spatial crowding and geometric constraints. These are implemented as probabilistic or threshold-based rules, each based on experimentally inferred functional forms, as described in §2.2 of the main text.

The probability of apoptosis through mechanical and contact-based competition is calculated every 10 Monte Carlo steps, which, on the timescale of the timer threshold, reproduces the experimentally observed probabilities. When a cell is eliminated through apoptosis, its target area is set to zero, causing it to shrink rapidly. During this process, surrounding cells grow to occupy the space left by the apoptotic cell. Within the simulation, this is implemented as:

(i) Iterate over each cell.
(ii) Calculate the local density for the given cell, $\rho_{c}$ (equation (2.4)) and determine the shared perimeter with cells of a different type.
(iii) Check whether the cell undergoes mechanical apoptosis with probability $P(\rho_{c})$ (equation (2.5)) or contact-based apoptosis (equation (2.6)).
(iv) If the cell is apoptotic, set the target area and elastic modulus to $A_{T}=0$ and λ=5, which would promote rapid, but not instantaneous, shrinkage.

In addition to apoptosis, cells may be eliminated via live-cell extrusions. This process occurs when a cell’s area decreases significantly relative to the average population area. Experimental observations [18] indicate that extrusion is triggered by compressive stress within the tissue, causing the cell to detach from the substrate and be ejected from the monolayer. In our simulations, any cell whose area falls below ⟨A⟩/4 (where ⟨A⟩ is the average area of the colony) is immediately eliminated, mimicking the rapid timescale of extrusion [18,75]. This is implemented in the simulation as follows:

(i) Calculate the average cellular area, ⟨A⟩, within the tissue.
(ii) Iterate over each cell and check whether its area, A, satisfies A<⟨A⟩/4. Any cell meeting this criterion is immediately ejected by converting its lattice sites into medium sites.

We note that our simplistic implementation of extrusion does not consider active processes—such as winner cell invasion or adhesion-dependent contractility—which may also trigger extrusion at higher cell areas, as suggested by recent experimental and theoretical studies [76,77]. Our current rule thus captures one mode of extrusion and could be generalized in future work.

## Appendix C. Model parametrization

To compare our simulations with physiological scenarios, converting lattice units in the CPM framework to physical units is essential. This involves two primary conversions: translating pixels to SI units for distance, and Monte Carlo steps (mcs) to SI units for time. To determine the distance conversion, we compared the experimentally reported average cell volume for MDCK cells during subconfluence [35,48,78] with the average subconfluent colony area in our simulations, ${\bar{A}}_{SC}=3200$ pixels. This yielded a conversion factor: 1 pixel =(0.13±0.03)µm^2^. Applying this factor to the G1/S size threshold gave us $A_{S}=1377$ pixels =(179.01±41.31) µm^2^. Based on this, we set the default sizer threshold to $A_{S}=1400$ pixels. We normalized simulation time with the average cell cycle duration in uncrowded conditions, i.e. the cell’s timer duration $\tau_{0}$. Experimental data for typical epithelial cells suggest that the S/G2/M phase lasts approximately $\tau_{0}\approx 10\;\text{h}$ [48,78]. This provides the timescale conversion from Monte Carlo steps to hours, $\tau_{0}=225\text{ mcs}\approx 10\text{ h}$ (or 1 mcs≈2.67 min).

The single-cell growth rate, G, is sampled from a Gaussian distribution and determines the change in target area per Monte Carlo step (equation (2.3)). The mean value, $G_{0}$, is set so that isolated cells maintain the average subconfluent area ${\bar{A}}_{SC}$, with $G_{0}=\frac{1}{2}\frac{{\bar{A}}_{SC}}{\tau_{0}}$. The standard deviation is set to 5% $G_{0}$. While G controls the growth rate ($\text{d}A^{T}/\text{d}t$) for isolated cells, crowding in the tissue restricts available space, causing the actual cell area to deviate from the target. This activates the exponential term in equation (2.3), $e^{-k(A-A^{T}{)}^{2}}$, driving $\text{d}A^{T}/\text{d}t$ *to* zero until the cell area increases. The parameter k, representing the sensitivity to crowding or contact inhibition of proliferation, quantifies the allowed deviation between the actual and target areas. The default value, k=0.01 pixels${,}^{-2}$, was empirically chosen to ensure appropriate colony growth dynamics over several cell cycles.

In our CPM model, five parameters define the energy scale in the Hamiltonian: the elastic energy term λ, and the contact energy terms $J_{\text{AA}}$, $J_{\text{BB}}$, $J_{\text{AB}}$ and $J_{\text{AM}}=J_{\text{BM}}=J_{\text{MM}}$. Here, λ represents the area elasticity modulus, set to a default value of λ=1. The J parameters represent the strength of intercellular adhesions; higher values of J indicate lower adhesion and higher surface tension. Accordingly, we set $J_{\text{AA}}=12.5$, $J_{\text{AB}}=10$, $J_{\text{BB}}=6$, and $J_{\text{AM}}=J_{\text{BM}}=J_{\text{MM}}=15$, allowing cells to adhere more strongly to each other than to the medium. The variations in $J_{\text{AA}}$, $J_{\text{BB}}$ and $J_{\text{AB}}$ resulted in different morphologies for the growing colony of type A cells.

We also tested the effect of polarized motility forces on mutant cells using the ExternalPotential plugin in CompuCell3D. Within the range of biologically relevant force magnitudes, we found that moderate values of active motility did not qualitatively alter competition outcomes (electronic supplementary material, figures S6, S7), indicating that mechanical and proliferative parameters dominate under the conditions studied.

## References

[1] Levayer R, Dupont C, Moreno E. 2016 Tissue crowding induces caspase-dependent competition for space. Curr. Biol. **26**, 670–677. (10.1016/j.cub.2015.12.072) 26898471 PMC4791483

[2] Vincent JP, Fletcher AG, Baena-Lopez LAl. 2013 Mechanisms and mechanics of cell competition in epithelia. Nat. Rev. Mol. Cell Biol. **14**, 581–591. (10.1038/nrm3639) 23942450

[3] Merino MM, Levayer R, Moreno E. 2016 Survival of the fittest: essential roles of cell competition in development, aging, and cancer. Trends Cell Biol. **26**, 776–788. (10.1016/j.tcb.2016.05.009) 27319281

[4] Moreno E. 2008 Is cell competition relevant to cancer? Nat. Rev. Cancer **8**, 141–147. (10.1038/nrc2252) 18185517

[5] Morata G, Ripoll P. 1975 Minutes: mutants of Drosophila autonomously affecting cell division rate. Dev. Biol. **42**, 211–221. (10.1016/0012-1606(75)90330-9) 1116643

[6] Moreno E, Basler K. 2004 dMyc transforms cells into super-competitors. Cell **117**, 117–129. (10.1016/s0092-8674(04)00262-4) 15066287

[7] Shraiman BI. 2005 Mechanical feedback as a possible regulator of tissue growth. Proc. Natl Acad. Sci. USA **102**, 3318–3323. (10.1073/pnas.0404782102) 15728365 PMC552900

[8] Matamoro-Vidal A, Levayer R. 2019 Multiple influences of mechanical forces on cell competition. Curr. Biol. **29**, R762–R774. (10.1016/j.cub.2019.06.030) 31386857

[9] Wagstaff L *et al*. 2016 Mechanical cell competition kills cells via induction of lethal p53 levels. Nat. Commun. **7**, 11373. (10.1038/ncomms11373) 27109213 PMC4848481

[10] Moreno E, Basler K, Morata G. 2002 Cells compete for decapentaplegic survival factor to prevent apoptosis in Drosophila wing development. Nature **416**, 755–759. (10.1038/416755a) 11961558

[11] Moreno E. 2013 Mechanisms of cell competition: themes and variations. J. Cell Biol. **200**, 689–698. (10.1083/jcb.201301051) 23509066 PMC3601360

[12] Levayer R, Hauert B, Moreno E. 2015 Cell mixing induced by myc is required for competitive tissue invasion and destruction. Nature **524**, 476–480. (10.1038/nature14684) 26287461

[13] Díaz-Díaz C, de Manuel LF, Jimenez-Carretero D, Montoya MC, Clavería C, Torres M. 2017 Pluripotency surveillance by myc-driven competitive elimination of differentiating cells. Dev. Cell **42**, 585–599. (10.1016/j.devcel.2017.08.011) 28919206

[14] Yamamoto M, Ohsawa S, Kunimasa K, Igaki T. 2017 The ligand Sas and its receptor PTP10D drive tumour-suppressive cell competition. Nature **542**, 246–250. (10.1038/nature21033) 28092921

[15] Basan M, Risler T, Joanny J, Sastre‐Garau X, Prost J. 2009 Homeostatic competition drives tumor growth and metastasis nucleation. HFSP J. **3**, 265–272. (10.2976/1.3086732) 20119483 PMC2799988

[16] Abercrombie M. 1979 Contact inhibition and malignancy. Nature **281**, 259–262. (10.1038/281259a0) 551275

[17] Puliafito A, Hufnagel L, Neveu P, Streichan S, Sigal A, Fygenson DK, Shraiman BI. 2012 Collective and single cell behavior in epithelial contact inhibition. Proc. Natl Acad. Sci. USA **109**, 739–744. (10.1073/pnas.1007809109) 22228306 PMC3271933

[18] Eisenhoffer GT, Loftus PD, Yoshigi M, Otsuna H, Chien CB, Morcos PA, Rosenblatt J. 2012 Crowding induces live cell extrusion to maintain homeostatic cell numbers in epithelia. Nature **484**, 546–549. (10.1038/nature10999) 22504183 PMC4593481

[19] Streichan SJ, Hoerner CR, Schneidt T, Holzer D, Hufnagel L. 2014 Spatial constraints control cell proliferation in tissues. Proc. Natl Acad. Sci. USA **111**, 5586–5591. (10.1073/pnas.1323016111) 24706777 PMC3992650

[20] Irvine KD, Shraiman BI. 2017 Mechanical control of growth: ideas, facts and challenges. Development **144**, 4238–4248. (10.1242/dev.151902) 29183937 PMC5769630

[21] Di Meglio I, Trushko A, Guillamat P, Blanch-Mercader C, Abuhattum S, Roux A. 2022 Pressure and curvature control of the cell cycle in epithelia growing under spherical confinement. Cell Rep. **40**, 111227. (10.1016/j.celrep.2022.111227) 36001958 PMC9433880

[22] Zhao B *et al*. 2007 Inactivation of YAP oncoprotein by the Hippo pathway is involved in cell contact inhibition and tissue growth control. Genes Dev. **21**, 2747–2761. (10.1101/gad.1602907) 17974916 PMC2045129

[23] Pan Y, Heemskerk I, Ibar C, Shraiman BI, Irvine KD. 2016 Differential growth triggers mechanical feedback that elevates Hippo signaling. Proc. Natl Acad. Sci. USA **113**, E6974–E6983. (10.1073/pnas.1615012113) 27791172 PMC5111668

[24] Kim NG, Koh E, Chen X, Gumbiner BM. 2011 E-cadherin mediates contact inhibition of proliferation through Hippo signaling-pathway components. Proc. Natl Acad. Sci. USA **108**, 11930–11935. (10.1073/pnas.1103345108) 21730131 PMC3141988

[25] Igaki T, Pastor-Pareja JC, Aonuma H, Miura M, Xu T. 2009 Intrinsic tumor suppression and epithelial maintenance by endocytic activation of Eiger/TNF signaling in Drosophila. Dev. Cell **16**, 458–465. (10.1016/j.devcel.2009.01.002) 19289090 PMC2729686

[26] Schroeder MC, Chen CL, Gajewski K, Halder G. 2013 A non-cell-autonomous tumor suppressor role for Stat in eliminating oncogenic scribble cells. Oncogene **32**, 4471–4479. (10.1038/onc.2012.476) 23108407

[27] Sun G, Irvine KD. 2011 Regulation of Hippo signaling by Jun kinase signaling during compensatory cell proliferation and regeneration, and in neoplastic tumors. Dev. Biol. **350**, 139–151. (10.1016/j.ydbio.2010.11.036) 21145886 PMC3038240

[28] Bove A, Gradeci D, Fujita Y, Banerjee S, Charras G, Lowe AR. 2017 Local cellular neighborhood controls proliferation in cell competition. Mol. Biol. Cell **28**, 3215–3228. (10.1091/mbc.e17-06-0368) 28931601 PMC5687024

[29] Gradeci D, Bove A, Charras G, Lowe AR, Banerjee S. 2020 Single-cell approaches to cell competition: high-throughput imaging, machine learning and simulations. Semin. Cancer Biol. **63**, 60–68. (10.1016/j.semcancer.2019.05.007) 31108201

[30] Nishikawa S, Takamatsu A, Ohsawa S, Igaki T. 2016 Mathematical model for cell competition: predator–prey interactions at the interface between two groups of cells in monolayer tissue. J. Theor. Biol. **404**, 40–50. (10.1016/j.jtbi.2016.05.031) 27234645

[31] Nishikawa S, Takamatsu A. 2019 Effects of cell death-induced proliferation on a cell competition system. Math. Biosci. **316**, 108241. (10.1016/j.mbs.2019.108241) 31449892

[32] Ranft J, Aliee M, Prost J, Jülicher F, Joanny JF. 2014 Mechanically driven interface propagation in biological tissues. New J. Phys. **16**, 035002. (10.1088/1367-2630/16/3/035002)

[33] Murphy RJ, Buenzli PR, Baker RE, Simpson MJ. 2020 Mechanical cell competition in heterogeneous epithelial tissues. Bull. Math. Biol. **82**, 130. (10.1007/s11538-020-00807-x) 32979100

[34] Tsuboi A, Ohsawa S, Umetsu D, Sando Y, Kuranaga E, Igaki T, Fujimoto K. 2018 Competition for space is controlled by apoptosis-induced change of local epithelial topology. Curr. Biol. **28**, 2115–2128.(10.1016/j.cub.2018.05.029) 29910075

[35] Gradeci D, Bove A, Vallardi G, Lowe AR, Banerjee S, Charras G. 2021 Cell-scale biophysical determinants of cell competition in epithelia. eLife (eds K Kruse, N Barkai, R Levayer), **10**, e61011. (10.7554/elife.61011) 34014166 PMC8137148

[36] Pak TF, Pitt-Francis J, Baker RE. 2024 A mathematical framework for the emergence of winners and losers in cell competition. J. Theor. Biol. **577**, 111666. (10.1016/j.jtbi.2023.111666) 37956955 PMC7618079

[37] Li X, Datta A, Banerjee S. 2024 Proliferation symmetry breaking in growing tissues. BioRxiv. (10.1101/2024.09.03.610990)

[38] Gupta P, Kayal S, Tanimura N, Pothapragada SP, Senapati HK, Devendran P, Fujita Y, Bi D, Das T. 2023 Mechanical imbalance between normal and transformed cells drives epithelial homeostasis through cell competition. Elife **14**, RP100967. (10.7554/eLife.100967.1)

[39] Bosveld F, Guirao B, Wang Z, Rivière M, Bonnet I, Graner F, Bellaïche Y. 2016 Modulation of junction tension by tumor-suppressors and proto-oncogenes regulates cell-cell contacts. Development **143**, 623–634. (10.1242/dev.127993) 26811379

[40] Lee SW, Morishita Y. 2022 Two types of critical cell density for mechanical elimination of abnormal cell clusters from epithelial tissue. PLoS Comput. Biol. **18**, e1010178. (10.1371/journal.pcbi.1010178) 35696420 PMC9232172

[41] Carpenter LC, Pérez-Verdugo F, Banerjee S. 2024 Mechanical control of cell proliferation patterns in growing epithelial monolayers. Biophys. J. **123**, 909–919. (10.1016/j.bpj.2024.03.002) 38449309 PMC10995431

[42] Graner F, Glazier JA. 1992 Simulation of biological cell sorting using a two-dimensional extended Potts model. Phys. Rev. Lett. **69**, 2013–2016. (10.1103/physrevlett.69.2013) 10046374

[43] Swat MH, Thomas GL, Belmonte JM, Shirinifard A, Hmeljak D, Glazier JA. 2012 Multi-scale modeling of tissues using CompuCell3D. In Methods in cell biology (eds AR Asthagiri, AP Arkin), pp. 325–366, vol. 110. Oxford, UK: Elsevier. (10.1016/b978-0-12-388403-9.00013-8) PMC3612985 22482955

[44] Rens EG, Edelstein-Keshet L. 2019 From energy to cellular forces in the cellular Potts model: an algorithmic approach. PLoS Comput. Biol. **15**, e1007459. (10.1371/journal.pcbi.1007459) 31825952 PMC6927661

[45] Metropolis N, Rosenbluth AW, Rosenbluth MN, Teller AH, Teller E. 1953 Equation of state calculations by fast computing machines. J. Chem. **21**, 1087–1092. (10.1063/1.1699114) 41342507

[46] Hastings WK. 2001 Monte Carlo sampling methods using Markov chains and their applications. Biometrika **57**, 97–109. (10.1093/oso/9780198509936.003.0015)

[47] Xie S, Skotheim JM. 2020 A G1 sizer coordinates growth and division in the mouse epidermis. Curr. Biol. **30**, 916–924. (10.1016/j.cub.2019.12.062) 32109398 PMC7158888

[48] Devany J, Falk MJ, Holt LJ, Murugan A, Gardel ML. 2023 Epithelial tissue confinement inhibits cell growth and leads to volume-reducing divisions. Dev. Cell **58**, 1462–1476.(10.1016/j.devcel.2023.05.018) 37339629 PMC10528006

[49] Bastounis EE *et al*. 2021 Mechanical competition triggered by innate immune signaling drives the collective extrusion of bacterially infected epithelial cells. Dev. Cell **56**, 443–460.(10.1016/j.devcel.2021.01.012) 33621492 PMC7982222

[50] Wullkopf L, West AKV, Leijnse N, Cox TR, Madsen CD, Oddershede LB, Erler JT. 2018 Cancer cells’ ability to mechanically adjust to extracellular matrix stiffness correlates with their invasive potential. Mol. Biol. Cell **29**, 2378–2385. (10.1091/mbc.e18-05-0319) 30091653 PMC6233061

[51] Xu W, Mezencev R, Kim B, Wang L, McDonald J, Sulchek T. 2012 Cell stiffness is a biomarker of the metastatic potential of ovarian cancer cells. PLoS One **7**, 1–12. (10.1371/journal.pone.0046609) PMC3464294 23056368

[52] Lin HH *et al*. 2015 Mechanical phenotype of cancer cells: cell softening and loss of stiffness sensing. Oncotarget **6**, 20946–20958. (10.18632/oncotarget.4173) 26189182 PMC4673241

[53] Fuhs T *et al*. 2022 Rigid tumours contain soft cancer cells. Nat. Phys. **18**, 1510–1519. (10.1038/s41567-022-01755-0)

[54] St. Croix B, Sheehan C, Rak JW, Flørenes VA, Slingerland JM, Kerbel RS. 1998 E-cadherin–dependent growth suppression is mediated by the cyclin-dependent kinase inhibitor p27^KIP1^. J. Cell Biol. **142**, 557–571. (10.1083/jcb.142.2.557) 9679152 PMC2133056

[55] McClatchey AI, Yap AS. 2012 Contact inhibition (of proliferation) redux. Curr. Opin. Cell Biol. **24**, 685–694. (10.1016/j.ceb.2012.06.009) 22835462

[56] Lekka M, Laidler P, Gil D, Lekki J, Stachura Z, Hrynkiewicz AZ. 1999 Elasticity of normal and cancerous human bladder cells studied by scanning force microscopy. Eur. Biophys. J. **28**, 312–316. (10.1007/s002490050213) 10394623

[57] Kiparaki M, Baker NE. 2023 Ribosomal protein mutations and cell competition: autonomous and nonautonomous effects on a stress response. Genetics **224**, d080. (10.1093/genetics/iyad080) PMC10691752 37267156

[58] Janiszewska M, Primi MC, Izard T. 2020 Cell adhesion in cancer: beyond the migration of single cells. J. Biol. **295**, 2495–2505. (10.1074/jbc.REV119.007759) PMC7039572 31937589

[59] Frieboes HB, Edgerton ME, Fruehauf JP, Rose FRAJ, Worrall LK, Gatenby RA, Ferrari M, Cristini V. 2009 Prediction of drug response in breast cancer using integrative experimental/computational modeling. Cancer Res. **69**, 4484–4492. (10.1158/0008-5472.can-08-3740) 19366802 PMC2720602

[60] Drasdo D, Höhme S. 2005 A single-cell-based model of tumor growth in vitro: monolayers and spheroids. Phys. Biol. **2**, 133–147. (10.1088/1478-3975/2/3/001) 16224119

[61] Li J, Schnyder SK, Turner MS, Yamamoto R. 2022 Competition between cell types under cell cycle regulation with apoptosis. Phys. Rev. Res. **4**, 033156. (10.1103/physrevresearch.4.033156)

[62] Ballesteros-Arias L, Saavedra V, Morata G. 2014 Cell competition may function either as tumour-suppressing or as tumour-stimulating factor in Drosophila. Oncogene **33**, 4377–4384. (10.1038/onc.2013.407) 24096487

[63] Bielmeier C, Alt S, Weichselberger V, La Fortezza M, Harz H, Jülicher F, Salbreux G, Classen AK. 2016 Interface contractility between differently fated cells drives cell elimination and cyst formation. Curr. Biol. **26**, 563–574. (10.1016/j.cub.2015.12.063) 26853359 PMC5282066

[64] Riehl BD, Kim E, Bouzid T, Lim JY. 2020 The role of microenvironmental cues and mechanical loading milieus in breast cancer cell progression and metastasis. Front. Bioeng. Biotechnol. **8**, 608526. (10.3389/fbioe.2020.608526) 33585411 PMC7874074

[65] Levayer R. 2020 Solid stress, competition for space and cancer: the opposing roles of mechanical cell competition in tumour initiation and growth. Semin. Cancer Biol. **63**, 69–80. (10.1016/j.semcancer.2019.05.004) 31077845 PMC7221353

[66] Alibert C, Goud B, Manneville J. 2017 Are cancer cells really softer than normal cells? Biol. Cell **109**, 167–189. (10.1111/boc.201600078) 28244605

[67] Hanahan D, Weinberg RA. 2011 Hallmarks of cancer: the next generation. Cell **144**, 646–674. (10.1016/j.cell.2011.02.013) 21376230

[68] Pachenari M, Seyedpour SM, Janmaleki M, Shayan SB, Taranejoo S, Hosseinkhani H. 2014 Mechanical properties of cancer cytoskeleton depend on actin filaments to microtubules content: investigating different grades of colon cancer cell lines. J. Biomech. **47**, 373–379. (10.1016/j.jbiomech.2013.11.020) 24315289

[69] Swaminathan V, Mythreye K, O’Brien ET, Berchuck A, Blobe GC, Superfine R. 2011 Mechanical stiffness grades metastatic potential in patient tumor cells and in cancer cell lines. Cancer Res. **71**, 5075–5080. (10.1158/0008-5472.can-11-0247) 21642375 PMC3220953

[70] Guck J *et al*. 2005 Optical deformability as an inherent cell marker for testing malignant transformation and metastatic competence. Biophys. J. **88**, 3689–3698. (10.1529/biophysj.104.045476) 15722433 PMC1305515

[71] Gal N, Weihs D. 2012 Intracellular mechanics and activity of breast cancer cells correlate with metastatic potential. Cell Biochem. Biophys. **63**, 199–209. (10.1007/s12013-012-9356-z) 22555560

[72] Cachoux VM. 2023 Epithelial apoptotic pattern emerges from global and local regulation by cell apical area. Curr. Biol. **33**, 4807–4826. (10.1016/j.cub.2023.09.049) 37827152 PMC10681125

[73] Carpenter LC, Banerjee S. 2025 BanerjeeLab/CellCompetition: cell competition model codes, version v1.0.0, 2025. Zenodo. (10.5281/zenodo.16915577)

[74] Carpenter LC, Banerjee S. 2025 Supplementary material from: Physical traits of supercompetitors in cell competition. Figshare. (10.6084/m9.figshare.c.8039813)41056994

[75] Kocgozlu L *et al*. 2016 Epithelial cell packing induces distinct modes of cell extrusions. Curr. Biol. **26**, 2942–2950. (10.1016/j.cub.2016.08.057) 27746027 PMC5423527

[76] Ogawa M, Kawarazaki Y, Fujita Y, Naguro I, Ichijo H. 2021 FGF21 induced by the ASK1-p38 pathway promotes mechanical cell competition by attracting cells. Curr. Biol. **31**, 1048–1057.(10.1016/j.cub.2020.11.052) 33357449

[77] Okuda S, Fujimoto K. 2020 A mechanical instability in planar epithelial monolayers leads to cell extrusion. Biophys. J. **118**, 2549–2560. (10.1016/j.bpj.2020.03.028) 32333862 PMC7231918

[78] Cadart C *et al*. 2018 Size control in mammalian cells involves modulation of both growth rate and cell cycle duration. Nat. Commun. **9**, 3275. (10.1038/s41467-018-05393-0) 30115907 PMC6095894

